# Prospects for Recyclable Multilayer Packaging: A Case Study

**DOI:** 10.3390/polym15132966

**Published:** 2023-07-06

**Authors:** Martina Seier, Vasiliki-Maria Archodoulaki, Thomas Koch, Bernadette Duscher, Markus Gahleitner

**Affiliations:** 1 Institute of Materials Science and Technology, TU Wien, Getreidemarkt 9, 1060 Vienna, Austria; vasiliki-maria.archodoulaki@tuwien.ac.at (V.-M.A.); thomas.koch@tuwien.ac.at (T.K.); 2 Borealis Polyolefine GmbH, Innovation Headquarters, St. Peter Str. 25, 4021 Linz, Austria; bernadette.duscher@borealisgroup.com (B.D.); markus.gahleitner@borealisgroup.com (M.G.)

**Keywords:** multilayer packaging, recycling, modified atmosphere, polyolefins, waste management

## Abstract

Food preservation is an essential application for polymers, particularly in packaging. Complex multilayer films, such as those used for modified atmosphere packaging (MAP), extend the shelf life of sensitive foods. These mostly contain various polymers to achieve the necessary combination of mechanic, optic, and barrier properties that limit their recyclability. As the European Union’s Circular Economy Action Plan calls for sustainable products and business models, including waste prevention policies and recycling quotas, with plastic packaging being a high priority, solutions towards more sustainable multilayer packaging are urgently needed. This study evaluated and compared the recycling potential of functionally equivalent PET (polyethylene terephthalate) and PP (polypropylene) post-consumer MAP through structure analysis and recycling simulation. The structure analysis revealed that both types of MAP contained functional (stability) and barrier layers (oxygen and moisture). The recycling simulation showed that the PP-based packaging was recyclable 10 times, maintaining its mechanical properties and functionality. At the same time, the PET-based MAP resulted in a highly brittle material that was unsuitable for reprocessing into similar economic value products. The secondary material from the PP-based MAP was successfully manufactured into films, demonstrating the functional possibility of closed-loop recycling. The transition from a linear to a circular economy for MAP is currently still limited by safety concerns due to a lack of sufficient and efficient purification methods, but the proper design of multilayers for recyclability is a first step towards circularity.

## 1. Introduction

Food preservation is one of the most common applications for polymers. Packaging accounts for approximately 44% of the global annual converter demand of 390.7 million tons [1]. Complex multilayer films, such as those used for modified atmosphere packaging (MAP), have a share of about 30% within this sector [2]. MAP is a sealed multilayer material system (consisting of a lid and a tray) that extends the shelf life of sensitive foods by creating a modified gas atmosphere. The longevity of the modified atmosphere is essential for its effectiveness. Therefore, the selected material must prevent gas diffusion and damage from external forces before content use [3]. A low water vapor transmission rate (WVTR) and oxygen transmission rate (OTR) are specifically required for this, typically requiring the combination of nonpolar and polar polymer elements.

Consequently, MAP may contain layers of up to nine or more different polymers, which are typically polyethylene (PE), polypropylene (PP), polyamide (PA), ethylene vinyl alcohol (EVOH), and polyethylene terephthalate (PET). The manufacturing process of multilayer films involves several steps, starting with the extrusion of virgin polymer resins. After extrusion, the polymer melt can be processed into films by blowing (tubular process) or casting (flat process). In order to form a multilayer structure, individual film layers can be combined in a molten (co-extrusion) or solid-state (lamination, with or without adhesives). Since most polymer melts cannot be easily combined due to structural differences, the use of adhesives (tie layers) and primers is necessary for the co-extrusion process. For surface finishing and decoration, MAP is commonly coated (glossy surface), printed (direct or reverse), or labeled (adhesive-attached paper or polymer labels) [4,5,6,7].

Those tightly assembled packaging structures, however, complicate the recycling process. Current mechanical recycling technologies are limited by their reliance on single-variety separation, making them unsuitable for the viable reprocessing of conventional MAP [8]. According to various studies on polymer streams [9,10] and recycling systems, this is one of the limiting factors for the necessary massive increase in plastic recycling.

While pure polyolefin [11] (PE and PP) and PET [12] streams can be recycled rather well, most MAP is, even if collected, currently ending up in the ‘reject’ streams of recycling units and incinerated for energy recovery. This is in contradiction with the European Union’s Circular Economy Action Plan (CEAP), adopted in March 2020 as a part of the “Green Deal” to make the EU (European Union) economy sustainable and reach the UN (United Nations) Sustainable Development Goals by 2030 [13,14]. The CEAP aims to make sustainable products and business models the norm, including waste prevention policies such as using recyclates in new products and recycling quotas of 55% in the packaging sector [15]. Plastic packaging is a high priority for circularity implementation and will be a focus for future actions and legislation.

Avoidance or substituting polymers with other materials is often considered a first-solution approach for reducing polymer waste. However, removing or replacing functional plastics from food packaging is only possible by sacrificing the numerous advantages and conveniences they offer [16]. Despite the broad scientific research effort in developing biobased materials for food packaging, their application is not yet viable due to both the lack of proper industrial recycling capacities [17,18] and because of a shortfall in performance. Using polymer substitutes, such as paper, aluminum, or glass, may quickly lead to higher transportation and manufacturing costs, not benefiting the carbon footprint or sustainability life cycle analysis [19,20,21].

However, there have been some promising recent research in certain packaging areas lately. Korte et al. [22] demonstrated that groundwood pulp and sugar cane trays demonstrated better preserving characteristics for tomatoes when compared to their other references, including rPET (recycling PET). Chen et al. [23] found that the recyclability of beverage-paper-based composites could be improved by extending producer responsibility, benefiting the recycling rate. Tsironi et al. [12] discussed the current trends regarding the future sustainability of PET bottles, and Sid et al. [24] identified bio-sourced polymers as an emerging alternative to conventional food packaging. The urgent need for a more comprehensive evaluation, as well as comparison tools, to assess the sustainability of packaging products and materials is also proposed in the literature [21,25].

Nevertheless, although polymer multilayer packaging is currently predominantly incinerated, it is still widely considered the most environmentally beneficial solution when compared to substitutes [26,27,28]. One key reason is the aforementioned balance between the mechanical performance required for logistics and handling, the thermal stability required for filling and/or thermal treatment, the optics required for customer appeal, and the barrier properties (WVTR and OTR) required for content preservation. In this respect, Table 1 presents an overview of these properties for one of the most frequently cited biobased and biodegradable polymers, poly(lactic acid) (PLA), as well as a number of conventional versions. PET films demonstrate high-temperature stability (melt temperature (T_m_): 256 °C) and moderate barrier against oxygen and moisture (OTR: 20.0 cm^3^/m^2^·d·bar, WVTR: 4.0 g/m^2^·d). PP has moderate temperature resistance (T_m_: 165 °C) and a poor oxygen barrier (OTR: 800.0 cm^3^/m^2^·d·bar), though it has an excellent moisture barrier (WVTR: 0.6 g/m^2^·d), while PLA has the lowest temperature resistance (T_m_: 146 °C) and a poor blocking function against oxygen and moisture (OTR: 280.0 cm³/m²·d·bar, WVTR: 14.0 g/m^2^·d). Only the multilayer combinations (PET/EVOH/PE, PP/EVOH/PE) result in the optimum balance of temperature resistance, stability, and barrier properties necessary for MAP films. One can easily see that the target property combination is further out of reach for PLA than for other polymers. Among other factors, this results from the very low crystallization speed [29] of this polymer, which makes the glass transition temperature (T_g_) the more relevant stability parameter. While PLA may be suitable for certain packaging applications, such as fresh produce that requires short shelf life and limited gas barrier requirements, its gas permeability, moisture barrier, and temperature sensitivity make it less suitable for modified atmosphere packaging.

This clearly indicates the need for recyclable polymer-based multilayer packaging, for which a critical analysis of the existing packaging recyclability (and the derivation of new structures from these results) will play a decisive role [33]. Recently, the research interest in the field of flexible packaging recycling has rapidly grown, highlighting the importance of advancing packaging sustainability [34,35,36,37,38,39]. The work is mainly focused on either the “design for” recycling (invention of novel packaging films) [40,41,42,43,44] or “design from” recycling (recycling possibilities [45] and recyclate application) [46,47,48,49] aspects but does not connect the topics for a holistic overview.

**Figure 1:**
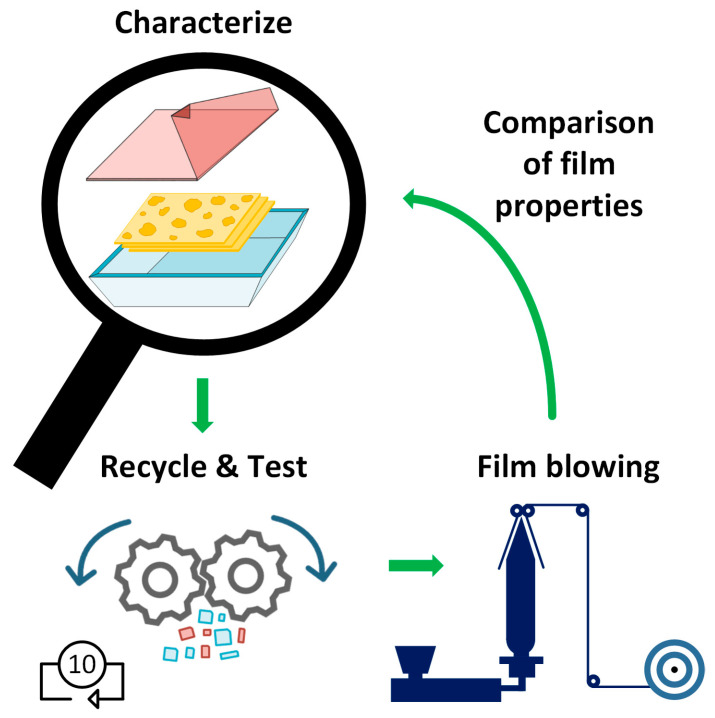
Research scheme.

This study was performed to evaluate the mechanical recycling potential of commercial PET- (majority) and PP-based (minority) post-consumer MAP, which is representative of the Austrian product market [50], and to connect the “design for” (packaging structure) and “design from” (assessment of recyclate properties and possible secondary applications) recycling aspects by following the scheme presented in Figure 1. It should be noted here that comparisons to other European countries, like Belgium [10], confirm the dominance of PP and PET in packaging waste streams (next to PE), adding to the logic of this choice.

The exact packaging structure (polymeric constituents, number of layers, and their thicknesses) of the individual components (lid or tray) was investigated to examine recycling (in combination with the results from secondary material testing) in terms of which packaging design structures are more or less suitable for mechanical recycling. Blends of the post-consumer MAP were processed following industrial procedures (cold-wash, grinding, and extrusion) [51] prior to the analysis of their mechanical, rheological, and morphological material properties, which were compared to each other. PP-based MAP (lid + tray) was further reprocessed up to 10 times (material samples were collected after 1, 3, 5, 7, and 10 reprocessing steps), tested mechanically (elastic modulus, elongation at break, and tensile impact toughness) and rheologically tested (melt flow rate, zero-shear viscosity, and extensional rheology) and was subsequently processed into new films using a micro blown-film line to simulate film-to-film closed-loop recycling. In order to highlight the functional possibility of the film-to-film recycling (possible secondary application) of PP-based MAP, the tensile modulus and puncture resistance of the blown films was tested and compared to virgin PP lids, which had similar thicknesses.

## 2. Materials and Methods

### 2.1. Materials and Test Specimen

PP (‘Berger’ ham)- and PET (‘Grana Padano’ cheese)-based post-consumer MAP was collected from household bins in eastern Austria to assess the recycling potential of the two different packaging products. Individual multilayer structures were analyzed and mechanically reprocessed (comparable to industrial practices), and properties, such as melt flow rate and stiffness, were tested and compared for material quality evaluation [51]. The recyclability of the system components was investigated by separate (lid or tray) and combined (lid + tray) reprocessing, according to Table 2. Currently, there is no uniform standard procedure for the mechanical recycling of polymer waste, but there are some existing guidelines, specifications, and pieces of research on possible certification systems for waste polymers, which provide guidance for processing and testing [52,53].

Prior to the mechanical recycling process, the MAP was sorted, cold-washed, and cut manually. A singled screw Extron extruder (EX-18-26-1.5, Extron Engineering Oy, Toijala, Finland) with a screw diameter of 18 mm and a length/diameter ratio of 25:1, equipped with three individual heating zones, was used for melt blending. Extrusion temperature was varied according to the packaging components based on the typical processing temperatures for the respective polymers (T_PP_: 220 °C for PP and T_PET_: 275 °C for PET-based packaging), and the screw speed was set to 70 rpm (this was found to give good homogeneity in earlier studies). The material strands from extrusion were shredded with a universal cutting mill (Pulverisette19, Fritsch, Idar-Oberstein, Germany) that had a 4 mm sieve inserted. For ‘PP Lid + Tray’, the process of extrusion and grinding was repeated up to 10 times (1×–10×, material samples were collected after 1, 3, 5, 7, and 10 reprocessing steps) to demonstrate the stability resp. degradation behavior of the PP-based MAP. The same reprocessing procedure could not be carried out for the PET-based packaging due to inferior material quality and liquid-like behavior after only one processing step.

### 2.2. Test Specimen

Test specimens for tensile and tensile impact testing, according to ISO 527-2 [54] and ISO 8256 [55], were produced by injection molding using a Haake Mini Lab II twin-screw extruder coupled with a Haake Mini Jet II injection molding unit (Thermo Fisher Scientific, Waltham, MA, USA). Extrusion temperature was set according to compounding temperature (T_PP_: 220 °C for PP and T_PET_: 275 °C for PET-based packaging); screw speed was 100 rpm, and mold temperature for injection was 40 °C (pressure: 350 bar, injection time: 10 s).

Specimens for dynamic shear and extensional rheology were produced by compression molding (Collin P 200 P, Maitenbeth, Germany) at a pressure of 100 bar and in line with extrusion temperatures of 220/275 °C. Discs with a diameter of 25 mm and a thickness of 1.2 mm and squares of 0.8 mm in thickness and a side length of 60 mm were generated using punched aluminum frames sandwiched between steel plates and were separated by Teflon^®^ sheets.

To demonstrate the possibility of film-to-film recycling, the recyclates were converted into 50 µm-thick films using an Ultra Micro blown-film line (LabTech Engineering, Samut Prakan, Thailand). Extrusion temperature was set to 200 °C; the die temperature was 180 °C; the fan for airflow cooling was set to 1700 rpm, and the pull-off speed of both roller sets was 1.1 m/min.

### 2.3. Multilayer Structure Analysis of Post-consumer MAP

The characterization of multilayer packaging structures was based on the user guide for the identification of polymers in multilayer films used in food contact materials released by the European Commission [56]. The inner and outer layers of post-consumer packaging were analyzed using a Bruker Tensor 27 Fourier-transform infrared (FT-IR) spectrometer (Billerica, MA, USA) with an attenuated total reflection (ATR) diamond (DuraSample IR II) and single reflection. A total of 16 scans with a 4 cm^−1^ resolution between 600–4000 cm^−1^ were conducted to identify the polymer spectra.

Differential scanning calorimetry (DSC) was used to detect all polymers within the outer layers included in the multilayer film structure by monitoring changes in heat flow attributed to physical changes such as melting [57]. Packaging samples were analyzed using a TA-Instruments TA Q2000 device (Newcastle, DE, USA) in a nitrogen atmosphere, with a sample mass of 5–6 mg and a temperature range of 0–300 °C, increasing at a rate of 10 K/min.

The thickness of each layer was determined using a Zeiss Axio Imager M2m light microscope (Oberkochen, Germany). Sections of 20 × 12 mm were cut from the post-consumer packaging and embedded with a two-component epoxy resin (Araldite AY103 + REN HY956) before being ground and polished with a Struers TegraForce-31 polisher (Copenhagen, Denmark). The percentual polymer mass proportion given in Table 1 was calculated using volumetric mass density (ρ_PET:_ 1.34 g/cm^3^, ρ_EVOH_: 1.16 g/cm^3^, ρ_PE_: 0.93 g/cm^3^, and ρ_PP_: 0.9 g/cm^3^) [58]. The combined mass proportions of the ‘Lid + Tray’ fractions resulted from the fact that the tray represents about 80 wt.% of the total packaging mass and the lid about 20 wt.% (detailed wt.% calculation can be found in the supplementary material).

### 2.4. Mechanical Properties of Recyclate Blends and Films

Specimens of 60 × 10 × 1 mm for the tensile impact strength test were notched on both sides using a Vis-Notch device (Instron, Darmstadt, Germany) and were tested with an Instron 9050 pendulum (equipped with 2 J hammer and 15 g crosshead mass; Instron, Darmstadt, Germany), according to ISO 8256/1A [55], to obtain the tensile impact strength a_tN_. Tensile testing was carried out in accordance with ISO 527-2-5A [54] and was performed on a universal testing machine (ZwickRoell Z050, Ulm, Germany) at a speed of 10 mm/min, equipped with a 1 kN load cell and an extensometer.

Mechanical testing of blown film properties was carried out in the tensile mode, according to ISO 527-3 [59], using strips measuring 100 × 10 mm (70 mm clamping length) and by puncture-resistance testing, performed according to DIN EN 14477 [60] on round samples at a crosshead speed of 10 mm/min.

### 2.5. Rheological Properties of Recyclate Blends

Dynamic shear rheology was performed via frequency sweeps on an MCR 302 rheometer (Anton Paar, Graz, Austria) equipped with a plate–plate system (1 mm gap size) and a nitrogen-purged heating hood. The temperature was held constant at 220 °C during the experiments, while deformation was raised logarithmically from 1% to 2% at a frequency range between 628 rad/s and 0.01 rad/s.

For extensional rheology measurements, the testing device was equipped with a CTD 450 heating chamber (nitrogen purged) and an SER-HPV 1 Sentmanat Extensional Rheometer (Xpansion instruments, Tallmadge, OH, USA). Test specimens were strained at three different rates (5 s^−1^, 1 s^−1^, and 0.1 s^−1^) at a temperature of 180 °C, in line with the film-blowing procedure. Base-line curves (LVE) were measured using a plate–plate system, using shear rates of 0.001 s^−1^ and 0.1 s^−1^.

Melt flow rate (MFR) measurements were performed according to ISO 1133 method A [61] under a load of 2.16 kg at 220/275 °C by using a manual testing device (MeltFloW basic, Karg Industrietechnik, Krailling, Germany).

### 2.6. Morphological Characterization of the Recyclate Blends

The morphology of the recycling blends that were recycled once was characterized using scanning electron microscopy (SEM) for the fractured tensile impact test specimen surfaces with an FEI Philips XL30 microscope (Hillsboro, OR, USA). Prior to imaging, the test samples were coated with gold (Agar Sputter Coater B7340, Essex, UK). Average particle size was estimated from an optical assessment of the fracture surfaces, considering the fact that this gives only a rough indication.

## 3. Results and Discussion

### 3.1. Multilayer Structure Analysis of Post-consumer PET- and PP-Based MAP Lids and Trays

**Figure 2:**
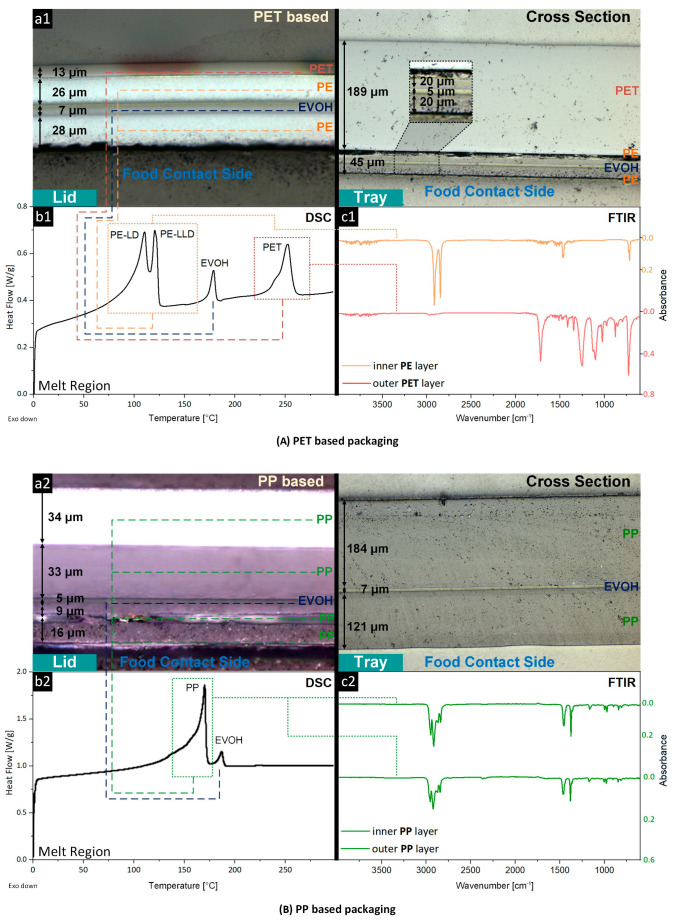
Packaging structure of PET (**a1**–**c1**)- and PP (**a2**–**c2**)-based MAP characterized by (**a**) light microscopy of film cross-sections, depicting the individual layers, (**b**) DSC (measurement of packaging film), revealing the melting peaks of the components, and (**c**) FT-IR spectroscopy (measurement of packaging film) for the identification of the inner (food contact) and outer layers. FT-IR and DSC curves of trays are provided in the Supplementary Materials (Figures S1–S4).

The multilayer analysis of the post-consumer MAP was realized through a combination of DSC (melt peak identification of all polymers in the multilayer film), FT-IR (identification of inner and outer multilayer film layers), and light microscopy (thickness of individual layers for mass content estimation through volumetric mass density), which is schematically presented in Figure 2. The analysis revealed that the PP-based lids and trays were thicker (lid: 97 µm; tray: 312 µm) when compared to the PET-based components (lid: 74 µm; tray: 234 µm). However, when having the same geometry, the PP packaging remains lighter due to its lower density of 0.9 g/cm^3^ when compared to the 1.34 g/cm^3^ of PET, which results in lower logistic costs.

The lids and trays of the PET-based MAP consisted of four layers, which can be divided into the barrier (food contact side) and carrier layers according to their functionality. The barrier layer for the lids and trays was similar, comprising one layer of oxygen-blocking EVOH (lid: 7 µm; tray: 5 µm) sandwiched between two layers of moisture-blocking PE (lid: 26/28 µm; tray: 20 µm) [7]. The DSC revealed that PE was present as a blend of PE-LD and PE-LLD (distinct melt peaks at 108 °C and 122 °C). A 13 µm-thin (lid) and 189 µm-thick (tray) PET layer were used to provide sufficient mechanical stability for the packaging against external damage such as punctures, tearing, or buckling [50]. The trays only contained removable paper labels but were otherwise transparent, while the lids were reverse printed. This technique prevents the ink from coming into direct contact with human skin or food products, as it is situated between the first and second film layers [62]. However, this makes the partial or complete removal of the color during the recycling process very difficult or even impossible, subsequently leading to the undesirable greyish coloring of the secondary material.

The PP-based MAP lids presented a five-layer structure with four PP layers (34/33/9/16 µm) and one layer of EVOH (5 µm), providing a barrier against oxygen. The PP was present as a mixture of PP copolymer (high toughness) and PP homopolymer (increased stiffness), estimated by the broad melting peak of PP between 145–160 °C, providing a sufficient moisture barrier and mechanical stability regarding the packaging. [7]. The trays only consisted of three layers: two thick layers of PP (184/121 µm) sandwiching one thin layer of EVOH (7 µm). The trays also contained a small portion of PE, which was observed in the DSC analysis, most likely functioning as a tie layer material for the co-extrusion of PP and EVOH [63]. Contrary to the PET-based lids, the PP-based lids were printed directly, allowing for the more efficient removal of the ink during the recycling process using flotation de-inking processes [64].

The lids and trays of the PET-based MAP consisted of similar polymers, but these polymers were present in different ratios (Table 1). The PET trays contained about 85 wt.% PET and 13 wt.% PE, while the lids contained only 23 wt.% PET and 66 wt.% PE. Polymer–polymer contamination plays an important role in the mechanical recycling process, as the mixture of highly incompatible polymers, such as PE and PET, leads to secondary materials of inferior quality when compared to virgin polymers [65]. The recyclate quality of the PET trays could be improved by using compatibilization agents [66] due to the lower PE contamination (13 wt.%) when compared to the lids (66 wt.%). However, when the PET lids and trays are combined to form a single packaging system, the recyclability and effectiveness of the compatibilization agents are limited (higher amounts of compatibilization agent are required) by increasing PE contamination (23 wt.%) when compared to the PET trays alone [67]. On the contrary, the PP-based MAP lids and trays contained >90 wt.% PP, resulting in reasonably homogeneous and, hence, more easily recyclable waste streams for both the individual components (lid or tray) and the entire packaging (lid + tray).

### 3.2. Comparison of the Property Profile of Reprocessed PET- and PP-Based MAP

Extrusion within the mechanical recycling process causes the inevitable formation of polymer blends from the inseparable layers. The more incompatible the individual components are to each other, the more unpredictable the resulting properties of the recyclate, subsequently making the design of and finding a suitable application for those materials very challenging [68] or requiring the use of a compatibilization agent.

**Figure 3:**
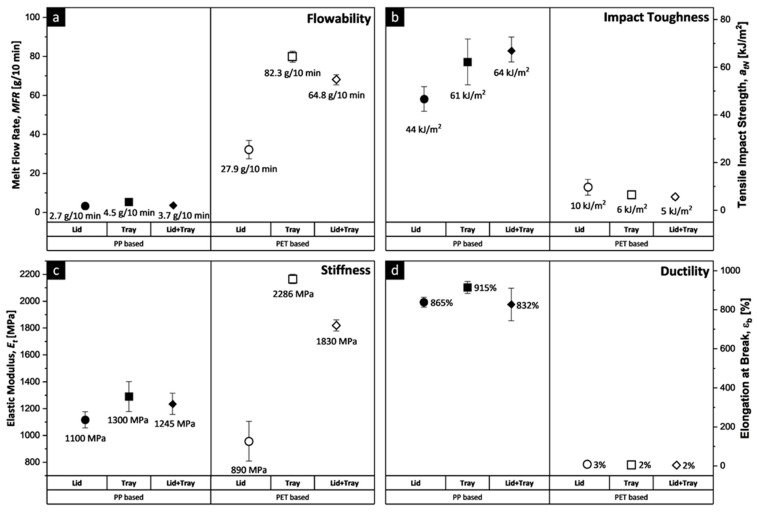
(**a**) Melt flow rate MFR [g/10 min], measured under a load of 2.16 kg at 220 °C (PP-based) and 275 °C (PET-based), (**b**) tensile impact strength a_tN_ [kJ/m^2^], (**c**) elastic modulus E_t_ [MPa], and (**d**) elongation at break ɛ_b_ [%] of one-time recycled PP (solid markers)- and PET (hollow markers)-based post-consumer MAP divided into individual components (lid or tray) and entire packaging (lid + tray).

Figure 3 compares the elastic modulus (E_t_), elongation at break (ε_b_), melt flow rate (MFR), and tensile impact strength (a_tN_) of recycled PET and PP-based post-consumer MAP, divided into individual system components (lid or tray) and entire packaging (lid + tray). In the material testing, the examined packaging types achieved very contrasting results. While the MFR for PP-based MAP varied between 2.7 g/10 min for the lids and 4.5 g/10 min for the trays, that of PET-based MAP was between 27.9 g/10 min for the lids due to the high PE content (66 wt.%), and 82.3 g/10 min for the trays. This leads to entirely different processing options regarding the secondary material, with MFR being the determining factor. PP-based MAP with an MFR of < 5 g/10 min offers the chance of being reprocessed into products of the same or similar economic value, such as films, while the high MFR of PET-based structures limits their applicability regarding injection molding [69], which is not the application the packaging film was originally designed for.

The tensile impact toughness of the PET-based MAP blends was extremely low, demonstrating values below 15 kJ/m^2^. Due to the relatively high fraction of PE (66 wt.%), which, in virgin form, demonstrates a_tN_ values of >150 kJ/m^2^, the PET lid had an a_tN_ of 10 kJ/m^2^, while this was halved for the trays. However, toughness is an essential characteristic of high-value polymer products such as packaging. Tough packaging ensures that the product is safe from damage or breakage during transportation, storage, and handling. It provides a layer of cushioning and absorbs shocks and vibrations that may occur during handling and transport [7]. The PP lids had a four times higher a_tN_ of 44 kJ/m^2^, and the PP trays presented an even higher value of 61 kJ/m^2^, which most likely resulted in a toughening effect from the presence of 5 wt.% PE within the trays, making the material a suitable source for secondary packaging applications.

The E_t_ values of polypropylene are strongly dependent on whether a homopolymer (up to 2000 MPa) or a copolymer is used and, in the case of the latter, also on how high the polyethylene content is, which decreases the modulus (between 800 MPa and 1400 MPa) [58]. The PP trays exhibited the highest E_t_ value of 1300 MPa, while the values for the PP lid + tray (1245 MPa) and PP lid (1100 Mpa) were only slightly lower. Virgin PET is stiffer compared to PP, presenting E_t_ values of about 3000 Mpa. For PET-based MAP recycling blends, the E_t_ ranged from 890 MPa for the lids (lowest PET content of 23 wt.%) to 2286 MPa for the trays (highest PET content of 85 wt.%).

Due to the mixture of the highly incompatible phases of PET and PE in PET-based MAP, the test specimen demonstrated brittle fracture behavior with ɛ_b_ values below 5%. The PP-based MAP blends were very ductile and exhibited ɛ_b_ values between 832% (lid + tray) and 915% (tray). The combined property profile (MFR, E_t_, ɛ_b,_ and a_tN_) clearly indicated that the recyclate from the PP-based MAP demonstrated higher recycling potential (low MFR, combined with moderate stiffness and high ductility and toughness) when compared to the recyclate from the PET-based MAP (moderate to high stiffness but high MFR and low ductility and toughness). In order to be considered economically recyclable, a material needs to retain its chemical and mechanical characteristics while being easily sortable by recycling companies. Since multilayers cannot simply be disassembled for recycling, the packaging design must ensure that they can be efficiently transformed and sorted at a reasonable cost without compromising their performance.

**Figure 4:**
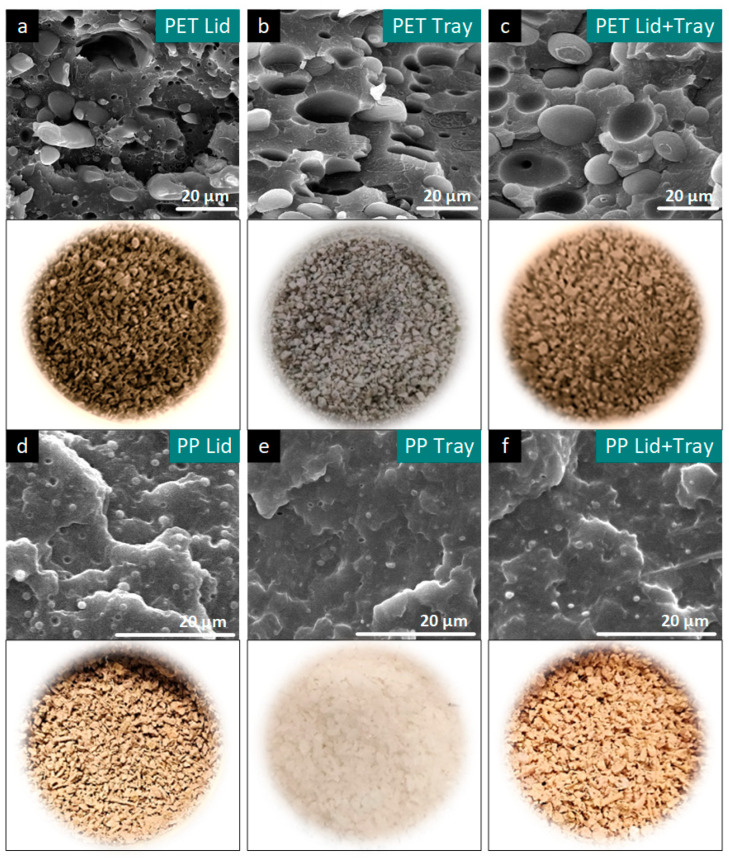
Samples of one-time recycled secondary materials and their morphologies, obtained from scanning electron microscopy of recycled (**a**–**c**) PET- and (**d**–**f**) PP-based post-consumer MAP divided into lids (**a**,**d**), trays (**b**,**e**), and lids + trays (**c**,**f**).

Samples of the recyclates, together with the scanning electron micrographs of the PET- and PP-based blends, are presented in Figure 4. For both the PET- and PP-based fractions, an apparent influence in color from the inks was observed even though different printing methods (reverse and direct) were used (no de-inking processes were part of the study). However, the directly printed (PP-based) blends resulted in lighter shades of beige, especially when compared to the dark brown PET-based trays. Besides the inks from printing, other contaminants, such as adhesives, sealants, or food residues, which migrate into the polymer matrix, contribute to the grey coloring and bad smell of recyclates [70]. Even though the recyclates from the PP-based MAP demonstrated a considerably more attractive appearance when compared to the PET-based MAP, coloring is not necessarily an indication of better or worse material performance. Hot- or cold-wash processes are frequently used in industrial waste treatment procedures to improve color and odor, but these are only ecologically viable if the secondary application demands low smell and inclusion content, as the mechanical parameters are hardly affected [71].

However, the morphology of a polymer blend, specifically its size, shape, and distribution of the dispersed phase, has a great influence on its performance. It affects properties such as mechanical strength or thermal stability [72]. The blends from the post-consumer PP-based MAP, which contained structurally similar blend partners, exhibited very fine morphologies and only relatively small particles (average < 5 µm), which were homogeneously distributed. No notable difference in particle size, particle size distribution, and surface condition between the morphologies of the PP lid, PP tray, and PP lid + tray blends was found. In contrast, the blends derived from the PET-based MAP demonstrated clear phase separation at the particle-matrix boundary and more coarse particles with average diameters of >20 µm, indicating the combination of highly incompatible phases (PET and PE) and a brittle structure confirmed by a ɛb of < 5% obtained from tensile testing. The blends from the PET trays and PET lids + trays, which contained predominantly PET (tray: 85 wt.%, lid + tray: 73 wt.%), had similar morphologies, comprising dispersed PE particles within a PET matrix (major component), while conversely, the PET lids containing 66 wt.% PE exhibited dispersed PET particles within a PE matrix (major component). Accordingly, the morphologies of the PET- and PP-based MAP recyclates are in good agreement with the mechanical and rheological properties.

### 3.3. Multiple Processing of the PP-Based MAP

#### 3.3.1. Mechanical Properties

In order to accomplish sustainable material circularity, it is essential to design products that can withstand not only single but multiple recycling procedures. In this case, not just the degree of organic and inorganic contaminants and compatibility of system components must be considered, but also how well that material (combination) can resist thermomechanical damage [73].

**Figure 5:**
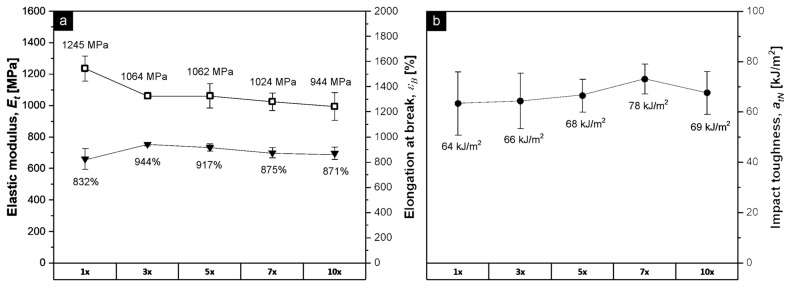
(**a**) E_t_ (hollow markers) and ɛ_b_ (solid markers) from tensile testing and (**b**) a_tN_ from tensile impact testing (injection molded specimen) of 10 times recycled PP-based MAP (lid + tray). The x-axis marks the number of reprocessing cycles.

Due to poor mechanical properties and a very high MFR, which led to liquid-like behavior, the repeated reprocessing of the PET-based MAP was not possible. In contrast to this, Figure 5 presents the E_t_, ɛ_b_, and a_tN_ after 1, 3, 5, 7, and 10 recycles of the post-consumer PP-based MAP (lids + trays). Within the 10 reprocessing steps, the E_t_ decreased by 25% (1245 MPa for the first recycling step, compared to 944 MPa for the tenth step), with the most significant drop occurring between the first and the third step (decreasing from 1245 MPa to 1064 MPa). This stiffness reduction is commonly seen in the degradation of PP and is normally explained as a result of reduced crystallinity and skin-layer orientation [74,75]. ɛ_b_ slightly increased from 832% for the first processing step up to 871% for the tenth step, which is in line with slightly reduced crystallinity. Again, the greatest change in values was observed between the first and the third step, with an increase of 13% from 832% to 944%. Tensile impact strength held relatively constant within the standard deviation during the 10 reprocessing steps, varying between 64 kJ/m^2^ (first step) and 69 kJ/m^2^ (tenth step). Even though there was a moderate drop in stiffness with increasing reprocessing steps, no embrittlement of the material was observed over 10 recycling cycles, with ductility and toughness remaining largely constant, indicating the good processing stability of the recyclates. However, these changes in mechanical properties could likely be reduced with the addition of processing stabilizers [76].

#### 3.3.2. Rheology

The high temperatures and shear forces associated with the mechanical recycling process of polymers result in increased chain scission related to changes in molecular weight, especially in the case of PP. The resulting change in molecular weight affects steady shear MFR, indirectly proportional dynamic shear viscosity values, and, subsequently, melt strength, which limits the reprocessing options [48]. The change in MFR and zero shear viscosity corresponds to an approximate reduction in average molecular weight from 400 to 300 kg/mol, which is a range where the effect on mechanical properties is only limited [77].

**Figure 6:**
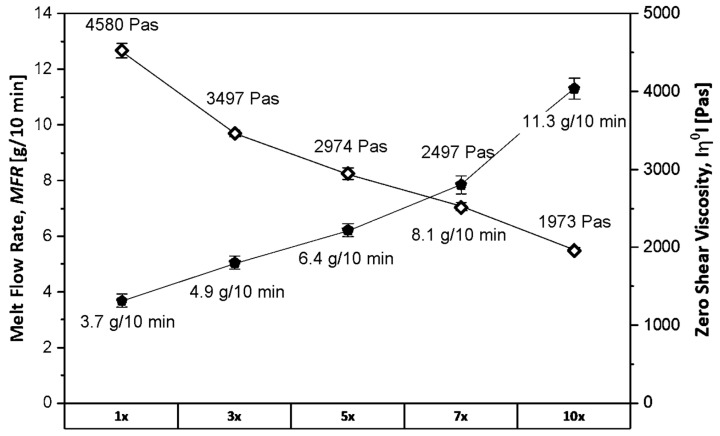
MFR (solid markers) and zero shear viscosity (hollow markers) of 10 times recycled PP-based MAP (lid + tray) measured under constant (2.16 kg load) and dynamic shear stress at 220 °C. The x-axis marks the number of reprocessing cycles.

During the 10 reprocessing steps of the PP-based post-consumer MAP (Figure 6), the MFR was gradually increased from 3.7 g/10 min (first step) to 11.3 g/10 min (tenth step) while, conversely, the zero-shear viscosity was decreased from 4580 Pas (first step) to 1973 Pas (tenth step). This behavior is typically reported for the re-extrusion of PP and is associated with material degradation and the β-scission of polymer chains. However, the percentual change of the measured values depends on the PP type, as homopolymers may demonstrate an MFR increase of 400% within five recycling cycles, while an increase of 100–150% in MFR is reported for PP copolymers [78,79]. This deviant degradation behavior may be explained by the branching or crosslinking reactions of the ethylene-containing polymer chains. Nevertheless, the major PP homopolymer portion will predominantly dictate the degradation process and, thus, the trend of MFR and viscosity during reprocessing [78]. In order to support those findings, crossover points (crossover modulus G_c_ and crossover frequency ω_c_) of storage (G′) and loss modulus (G″) from the dynamic shear rheology measurements are presented in Table 3.

The crossover point indicates the transition from predominantly elastic to viscous behavior regarding the polymer melt. A shift in the crossover point can be associated with changes in molecular weight (M_W_) and molecular weight distribution (MMD) [80,81,82]. The location of the crossover point at higher frequencies indicates the presence of shorter or broken polymer chains (shorter relaxation times), while a shift to higher frequencies indicates longer chains or branching reactions. Conversely, a shift to higher G_c_ values is related to a broadening of the MMD and vice versa for a shift to lower values [83]. For the 10 times recycled PP-based lid + tray blends, a clear shift in ω_c_ to higher values (45 rad/s to 82 rad/s) and, subsequently, a loss in M_w_ indicates chain scission through thermomechanical degradation during the individual reprocessing steps, which was observed. However, the highest MFR after 10 processing cycles of 11 g/10 min is still low enough for the production of films from the recyclate [84].

**Figure 7:**
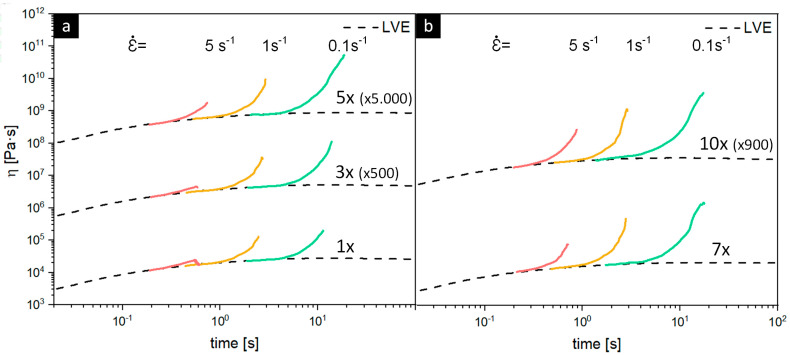
Vertically shifted extensional rheology curves of 10 times recycled (**a**,**b**) PP-based MAP (lid + tray) measured at 180 °C with strain rates of 5 s^-1^ (red lines), 1 s^-1^ (yellow lines), and 0.1 s^-1^ (green lines).

The stress resistance to the uniaxial extension of polymer melts is closely related to chain length and the degree of branching, which is visualized in Figure 7, which demonstrates the extensional rheology curves of the 10 times reprocessed PP-based MAP [85]. Good process stability and the output of high-quality films can only be realized with high melt strength polymers due to their relatively high extensional shear forces, to which PP homopolymers usually do not belong as a result of their linear chain structure [86]. Only the presence of ethylene in the copolymers results in the strain hardening of the melt, which was observed for all analyzed post-consumer PP MAP blends, especially at the lower strain rates of 0.1 and 1 s^−1^. While all blends exhibited pronounced strain hardening for all processing steps at shear rates of 0.1 and 1 s^−1^, for a strain of 5 s^−1^, the strain hardening effect gradually increased from the fifth up to the tenth reprocessing step, indicating branching reactions within the ethylene phase [87]. The extensional rheology measurement has shown that the melt strength of the recyclates is sufficient for application in processes that require high extensional viscosities, such as film blowing (possible design from recycling application).

### 3.4. Film Blowing of Reprocessed PP-Based MAP

**Figure 8:**
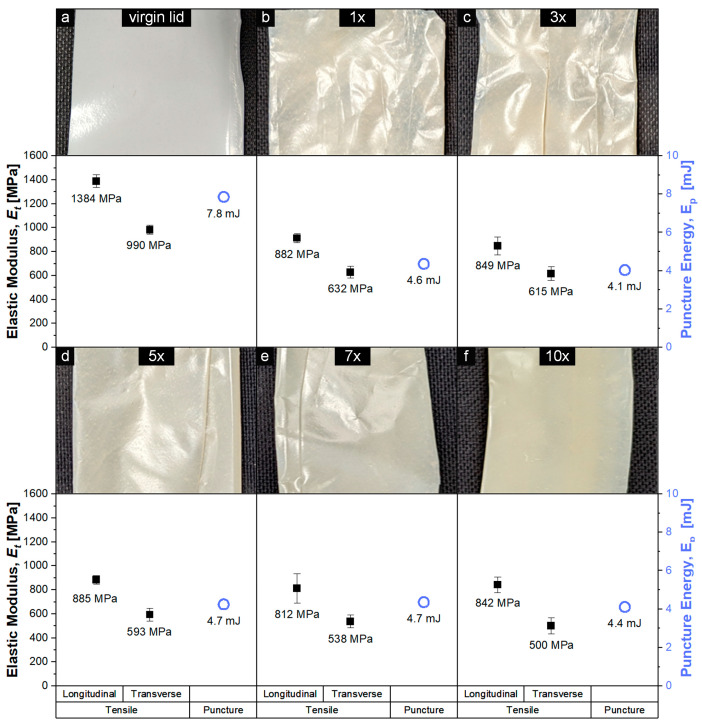
E_t_ from tensile and puncture energy and E_p_ from puncture-resistance testing (blown film) of 10 times recycled PP-based MAP (lid + tray) with a thickness of 50 µm (**b**–**f**), compared to the virgin packaging lid with a thickness of 100 µm (**a**).

In order to support the assumptions from the extensional rheology measurements and to demonstrate the application possibilities of the recyclates, the reprocessed PP-based MAP was manufactured into films, and its appearance and mechanical properties (elastic modulus and puncture energy) were compared to the lid of the original PP-based packaging. Figure 8 depicts blown-film samples with a thickness of 50 µm from 1, 3, 5, 7, and 10 times reprocessed PP-based MAP (Figure 8b–f), accompanied by the elastic modulus E_t_ (longitudinal and transverse film direction) and puncture energy E_p_ from the mechanical film testing, as compared to the virgin PP packaging lid (Figure 8a) with a thickness of 100 µm. All recycling blends could be successfully manufactured into films, which confirmed the results from the extensional rheology measurements. The films were opaque and presented a light beige coloring comparable to the color of the recyclate granules and included small (<0.5 mm) spots formed from contaminants or branched gels.

E_p_ remained rather constant within the 10 reprocessing steps, demonstrating a mean average value of 4.5 mJ. This was 3.3 mJ lower when compared to the virgin MAP lid, but as the parameter is increased linearly via film thickness (virgin lid: 100 µm; recycled film: 50 µm), puncture resistance widely remained the same during the recycling procedure. E_t_, which is not affected by film thickness but processing parameters, such as blow-up ratio or film orientation, was about 35% lower in both measurement directions after the first recycling step (longitudinal: 882 Mpa; transverse: 632 MPa) when compared to virgin material (longitudinal: 1384 MPa, transverse: 990 MPa). From the first to the tenth processing step, E_t_ decreased only slightly in the longitudinal measurement direction from 882 MPa to 842 MPa and from 632 MPa to 500 MPa in the transverse measurement direction, which is in agreement with the approximated molecular weight loss [77]. The mechanical properties of the recycling films were comparable to the original PP-based packaging film properties, which demonstrates the possibility of film-to-film recycling and, therefore, the application of the recyclate in secondary products similar to the purpose they were originally designed for. Currently, food-grade to food-grade recycling is limited by safety concerns regarding the migration or formation of harmful substances during the recycling process, but nonetheless, the recyclates could be used as a middle layer in a multilayer structure to reduce virgin material use [88].

## 4. Conclusions

To date, most MAP is, even if collected, currently ending up in the ‘reject’ streams of recycling units and is incinerated for energy recovery. The leap from a linear to a circular economy for MAP can only succeed if the packaging can be recycled into products of similar economic value (film-to-film recycling). Therefore, repeated film-to-film recycling and the maintenance of film properties over more than one reprocessing step offer great potential to approach circularity and establish multilayers as a valuable source of material in the future.

The prospects for the mechanical recycling of comparable (purpose and shelf-life) commercial post-consumer PET- and PP-based MAP were evaluated via a structural comparison (analyzed using FT-IR, DSC, and cross-section micrographs) of the components (lid or tray) and recycling simulation (cold-wash, grinding, and extrusion), followed by material testing (MFR, E_t_, ɛ_b_, and a_tN_).

In the first step, the compatibility of the components was investigated. The structure analysis revealed that both PET- and PP-based MAP comprised functional layers that provided stability (PET or PP homopolymer) and barrier layers (PE/PP-EVOH-PE/PPE) to block oxygen or moisture. The PET-based MAP, which contained highly incompatible PET and PE, resulted, however, in highly brittle material (*MFR*: 27.9–82.3 g/10 min; *E_t_*: 890–2286 Mpa; ɛ_b_: 2–3%, *a_tN_*: 5–10 kJ/m^2^), which is, subsequently, not suitable for products of similar economic value to packaging films. In contrast, the secondary material from the PP-based lids and trays exhibited a property profile closely resembling virgin PP (*MFR*: 2.7–4.5 g/10 min; *E_t_*: 1100–1300 Mpa; ɛ_b_: 832–915%; *a_tN_*: 44–64 kJ/m^2^).

In a further step, the ability to recycle was investigated. Due to the poor mechanical properties and difficult processability (high *MFR*) of PET-based MAP, the repeated reprocessing of this waste stream was not possible. However, the PP-based packaging (lid + tray) was recycled 10 times to demonstrate recycling capability and remained widely intact as the *MFR* increased from 3.7 g/10 min to 11.3 g/10 min, the *E_t_* decreased from 1245 MPa to 944 Mpa, and ɛ_b_ and a_tn_ were maintained within the standard deviation at average values of 888% and 69 kJ/m^2^. Good melt strength (pronounced strain hardening at strain rates of 0.1 and 1 s^−1^) for the PP-based MAP after being reprocessed 10 times was estimated by extensional rheology measurements and was confirmed by film-blowing experiments on a micro lab unit.

Even though the manufactured films did not have a flawless surface, for which a high degree of purification that would demand the consumption of resources (energy, chemicals, and water) would have been necessary prior to processing, the appearance had little to no effect on the mechanical properties and, thus, functionality, which allows for its reuse as a polymer film for applications such as secondary packaging.

The PP-based MAP has clearly demonstrated its superiority in terms of “design for” and “design from” recycling characteristics when compared to the PET-based MAP, which is, unfortunately, the predominant packaging source in the Austrian market. These findings make the structure design of PP-based MAP an attractive prospect to improve mechanical recycling rates within this packaging segment (mandatory contribution to the EU recycling targets) to prevent incineration, which is the common end-of-life treatment for multilayer films that combine PET and PE.

## Figures and Tables

**Table 1:** Comparison of elastic modulus (E_t_), glass transition temperature (T_g_, main load-bearing layer), melt temperature (T_m_, main load-bearing layer), oxygen transmission rate (OTR), and water vapor transmission rate (WVTR) for conventional packaging films compared to PLA (poly(lactic acid)). * Normalized to 100 µm in thickness [30,31,32].

Film Sample	E_t_ [MPa]	T_g_ [°C]	T_m_ [°C]	OTR [cm³/m²·d·bar]	WVTR [g/m²·d]
PET	3021	70	256	20.0 *	4.0 *
PP	1530	0	165	800.0 *	0.6 *
PLA	3600	55	146	280.0 *	14.0 *
PET/PE	2851	70	256	18.6	1.3
PET/EVOH/PE	3395	70	256	1.5	1.1
PP/EVOH/PE	2368	0	165	0.4	0.2

**Table 2:** Composition of investigated post-consumer polypropylene (PP)- and polyethylene terephthalate (PET)-based blends from modified atmosphere packaging (MAP) representing the disassembled (lid or tray) and combined packaging (lid + tray) structure processed at T_PP_ = 220 °C (PP-based packaging) or T_PET_ = 275 °C (PET-based packaging).

Composition (Estimated from Analysis)				Blend Model	Extrusion		
EVOH	PE- LD/LLD	PET	PP	Representation	T_PET_	T_PP_	Processing Steps
4 wt.%	23 wt.%	73 wt.%	-	‘PET Lid + Tray’	275 °C	-	1×
2 wt.%	13 wt.%	85 wt.%	-	‘PET Tray’	275 °C	-	1×
11 wt.%	66 wt.%	23 wt.%	-	‘PET Lid’	275 °C	-	1×
4 wt.%	4 wt.%	-	92 wt.%	‘PP Lid + Tray’	-	220 °C	10×
3 wt.%	5 wt.%	-	92 wt.%	‘PP Tray’	-	220 °C	1×
6 wt.%	-	-	94 wt.%	‘PP Lid’	-	220 °C	1×

**Table 3:** Results of crossover point (G′ = G″) expressed as crossover frequency ω_c_ and crossover modulus G_c_ from dynamic shear rheology measurements at 220 °C of 10 times recycled PP-based MAP (lid + tray). Allowing the estimation of higher (↑)/lower (↓) molecular weight (M_W_) and molecular weight distribution (MMD).

Sample	ω_c_ [rad/s]	G_c_ [Pa]	M_W_/MMD
PP lid + tray 1×	45	31,230	
PP lid + tray 3×	59	31,522	M_w_ ↓, MMD ↑
PP lid + tray 5×	64	31,610	M_w_ ↓, MMD ↑
PP lid + tray 7×	73	31,789	M_w_ ↓, MMD ↑
PP lid + tray 10×	82	31,816	M_w_ ↓, MMD ↑

## Data Availability

The data presented in this study are available on request from the corresponding author.

## Supplementary Materials

The following supporting information can be downloaded at: https://www.mdpi.com/article/10.3390/polym15132966/s1, Detailed wt.% calculation; Figure S1: FT-IR spectra of inner and outer PET tray layers.; Figure S2: DSC melt region of PET tray.; Figure S3: FT-IR spectra of inner and outer PP tray layers.; Figure S4: DSC melt region of PP tray.
