# Supplementary material for: Prospects for Recyclable Multilayer Packaging: A Case Study
Source: Polymers (Basel). 2023 Jul 6;15(13):2966. doi: 10.3390/polym15132966 (PMC10346915; doi:10.3390/polym15132966)
Supplement: Supplementary file 1 [file polymers-15-02966-s001.zip › polymers-2445923-supplementary.pdf]

Detailed wt.% calculation:

Mass content estimation (additional data for table 1) – Calculation example for **PET tray** :

Area of cut film sample: 9 cm^2

Mass of cut film sample: 0.285 g

PET proportion:	Thickness PET layer: 0.0189 cm
	Volume of PET layer: 0.1701 cm^3
	Density of PET: 1.34 g/cm^3
	**PET mass tray**: 0.227 g → 80 wt.%
PE proportion:	Thickness PE layers: 0.004 cm
	Volume of PE layers: 0.036 cm^3
	Density of PE: 0.93 g/cm^3
	**PE mass tray**: 0.0334 → 12 wt.%
EVOH proportion:	Thickness EVOH layer: 0.0005 cm
	Volume of EVOH layer: 0.0045 cm^3
	Density of EVOH: 1.16 g/cm^3
	**EVOH mass tray**: 0.005 g → 2 wt.%

The remaining mass of 0.0196 g (6 wt.%) which refers to adhesives and measurement errors is split into the individual polymer sections (PET, PE, EVOH) according to their percentage for simplification which results in the overall polymer mass estimation of

PET: 85 wt.%
PE: 13 wt.%
EVOH: 2 wt.%

for the PET tray.

For further simplification, the samples were not weighed, but the mass fractions directly calculated from the layer thicknesses.

PET tray:

PET proportion: $0.0189\ cm * 1\ cm^3 * 1.34 \frac{g}{cm^3} = 0.0253\ g$

PE proportion: $0.004\ cm * 1\ cm^3 * 0.93 \frac{g}{cm^3} = 0.00372\ g$

EVOH proportion: $0.0005\ cm * 1\ cm^3 * 1.16 \frac{g}{cm^3} = 0.00058\ g$

Total mass: 0.0296 g → **PET:** 85 wt.%, **PE:** 13 wt.%, **EVOH:** 2 wt.%

PET lid:

PET proportion: $0.0013\ cm * 1cm^3 * 1.34 \frac{g}{cm^3} = 0.001742\ g$

PE proportion: $0.0054\ cm * 1cm^3 * 0.93 \frac{g}{cm^3} = 0.005022\ g$

EVOH proportion: $0.0007\ cm * 1\ cm^3 * 1.16 \frac{g}{cm^3} = 0.000812\ g$

Total mass: 0.007576 g → **PET:** 23 wt.%, **PE:** 66 wt.%, **EVOH:** 11 wt.%

PP tray:

PP proportion: $0.0305\ cm * 1cm^3 * 0.9 \frac{g}{cm^3} = 0.02745\ g$

EVOH proportion: $0.0007\ cm * 1\ cm^3 * 1.16\ g/cm^3$= 0.000812 g

Total mass: 0.028266 g → **PP:** 97 wt.% , **EVOH:** 3 wt.%

Estimation of PE content in PP tray through melt enthalpy difference of PP peak in lid and tray DSC curves:

Melt enthalpy PP lid: 78 J/g

Melt enthalpy PP tray: 74 J/g

PP lid:

PP proportion: $0.0092\ cm * 1\ cm^3 * 0.9 \frac{g}{cm^3} = 0.00828\ g$

EVOH proportion: $0.0005\ cm * 1\ cm^3 * 1.16 \frac{g}{cm^3} = 0.00058\ g$

Total mass: 0.00886 g → **PP:** 94 wt.%, **EVOH:** 6 wt.%

FT-IR and DSC PET-based tray (additional data for figure 2):

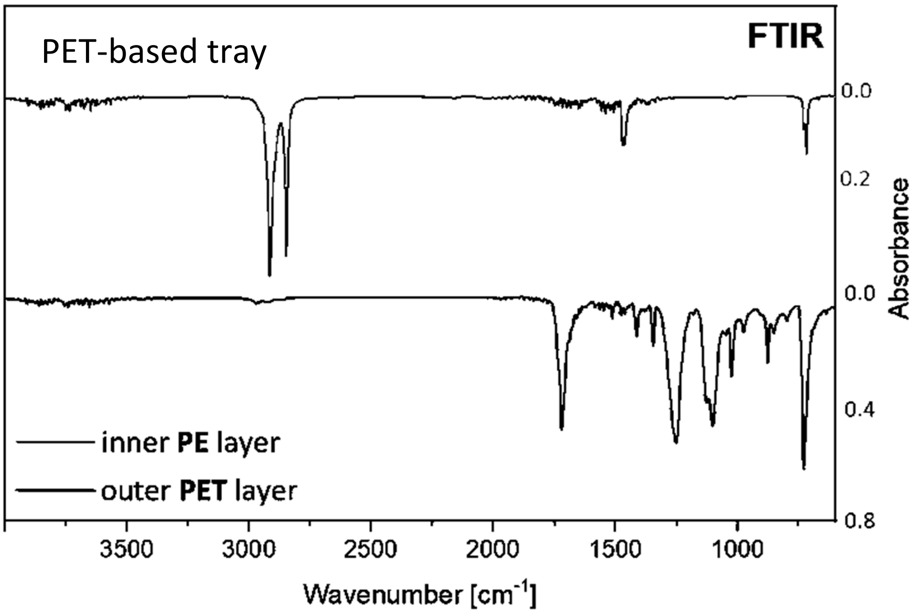

Figure S1. FT-IR spectra of inner and outer PET tray layers.

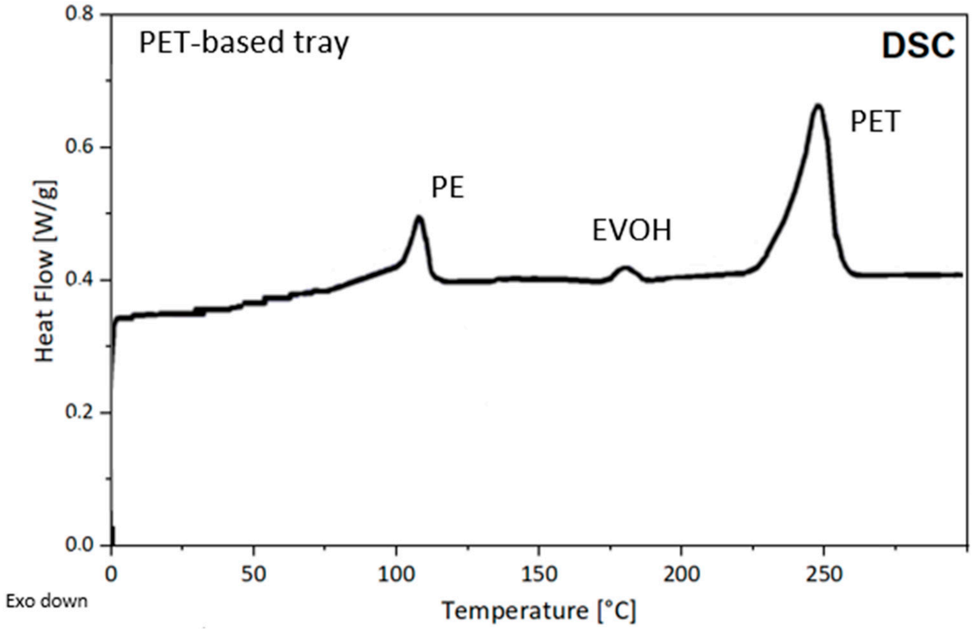

Figure S2. DSC melt region of PET tray.

FT-IR and DSC PP-based tray (additional data for figure 2):

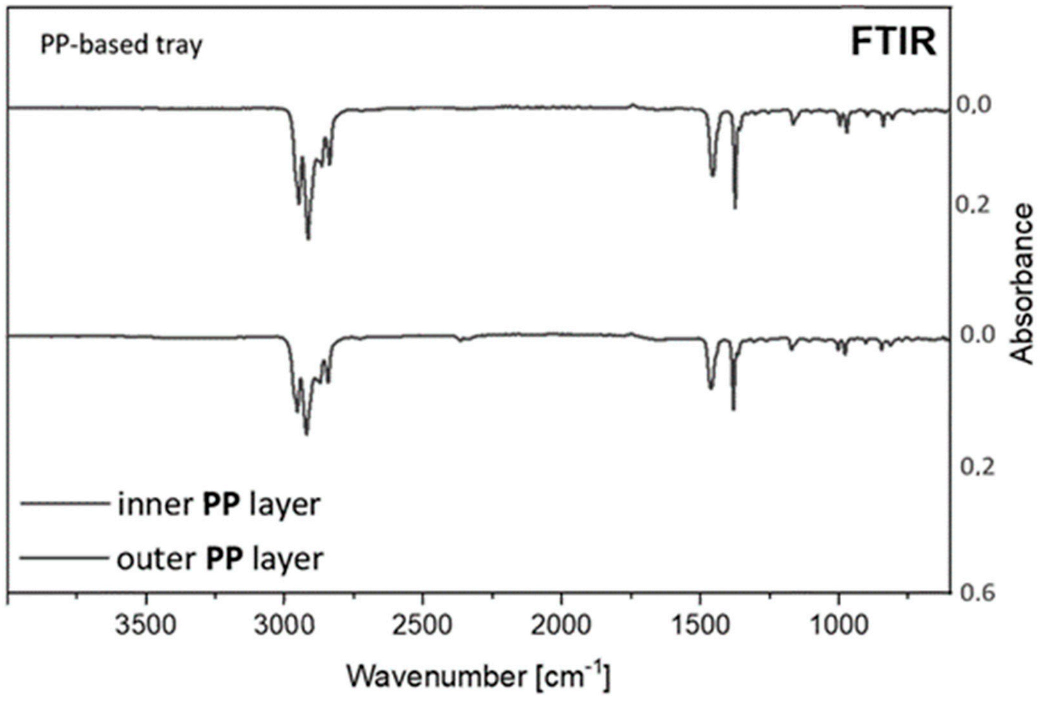

Figure S3. FT-IR spectra of inner and outer PP tray layers.

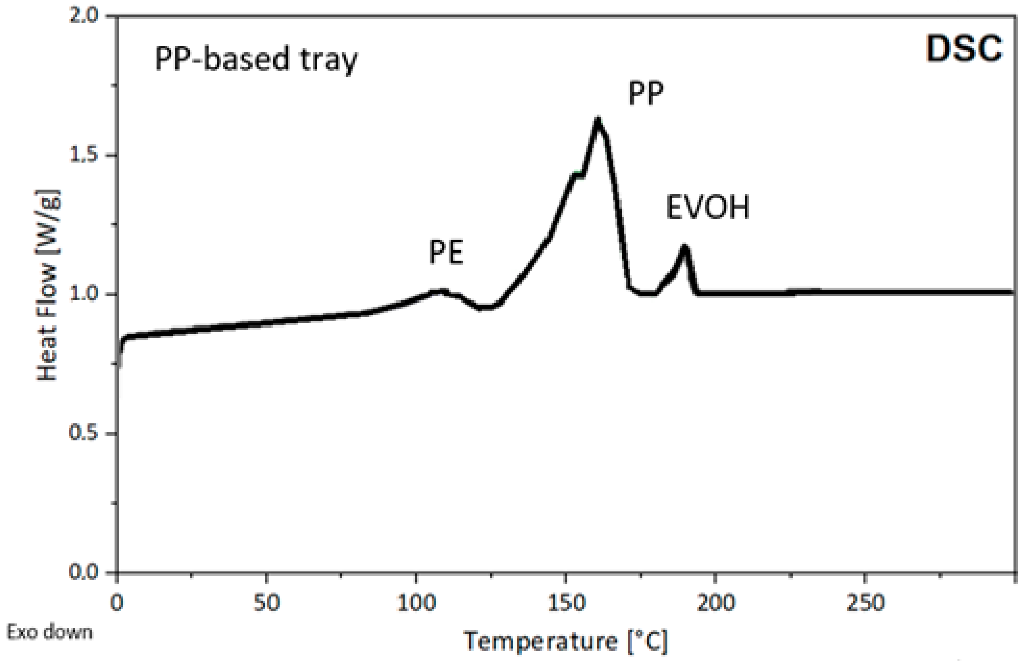

Figure S4. DSC melt region of PP tray.
